# A train the trainer program for healthcare professionals tasked with providing psychosocial support to breast cancer survivors

**DOI:** 10.1186/s12885-017-3965-2

**Published:** 2018-01-06

**Authors:** Eunyoung Park, Junghee Yoon, Eun-kyung Choi, Im Ryung Kim, Danbee Kang, Se-Kyung Lee, Jeong Eon Lee, Seok Jin Nam, Jin Seok Ahn, Adriaan Visser, Juhee Cho

**Affiliations:** 1 College of Nursing, Chungnam National University, Daejeon, South Korea; 2 Department of Digital Health, SAIHST, Sungkyunkwan University, Seoul, South Korea; 3 Cancer Education Center, Samsung Comprehensive Cancer Center, Samsung Medical Center, Sungkyunkwan University School of Medicine, Seoul, South Korea; 4 Department of Clinical Research Design and Evaluation, SAIHST, Sungkyunkwan University, 81 Irwon-ro, Gangnam, Seoul, 06351 South Korea; 5 Division of Breast and Endocrine Surgery, Department of Surgery, Samsung Medical Center, Sungkyunkwan University School of Medicine, Seoul, South Korea; 6 Division of Hematology/Oncology, Department of Medicine, Samsung Medical Center, Sungkyunkwan University School of Medicine, Seoul, South Korea; 7 PRO-Health, Rotterdam, the Netherlands; 8 Department of Epidemiology and Health, Behavior and Society, Johns Hopkins Bloomberg School of Public Health, Baltimore, MD USA

**Keywords:** Program development, Program evaluation, Breast cancer survivors, Healthcare providers, Psychosocial support, Professional education

## Abstract

**Background:**

The objective of this study is to develop, implement, and evaluate a training program for healthcare providers to improve ability to provide psychosocial support to breast cancer survivors in Korea.

**Methods:**

Based on a needs assessment survey and in-depth interviews with breast cancer survivors, a multidisciplinary team developed two-day intensive training program as well as education materials and counseling notes. Participants’ overall satisfaction was evaluated after the training.

**Results:**

The training program included a total of 16 lectures held over the course of seven sessions. Forty-one nurses and 3 social workers participated in the training program. Mean age was 37.5(± 6.4) years, and on average, they had 11.1 (± 5.6) years of experience. Participants’ overall satisfaction was good as following: program contents (4.04), trainee guidebook (3.82), location and environment (4.10), and program organization (4.19). Among the participants, 31 (70.4%) received certification after submitting real consultation cases after the training.

**Conclusion:**

Two day intensive training can provide a comprehensive and coordinated education to healthcare professionals for implementing survivorship care with an emphasis on psychosocial support. Furthermore, the program should resume as a periodic continuing education course for healthcare providers. Similar education for graduate students in oncology nursing would be beneficial.

**Electronic supplementary material:**

The online version of this article (10.1186/s12885-017-3965-2) contains supplementary material, which is available to authorized users.

## Background

Breast cancer is the most common cancer for women worldwide [1] and the fifth leading cause of cancer death for Korean women [2]. In recent years, early detection and effective treatment has led to more women surviving breast cancer, which has raised awareness of issues related to breast cancer survival [1, 3]. Despite advances in medicine intended to treat the physical components of the disease, psychosocial support for breast cancer survivors is still inadequate [4, 5]. Similarly, Korean breast cancer survivors reported challenges and difficulties in life following treatment and indicated a need for psychosocial support [6]. Yet, women with breast cancer often suffer from a lack of information and emotional support, both during the acute phase of the disease and beyond [3, 7].

While there are several guidelines for healthcare professionals regarding management of physical symptoms of cancer patients, there is only limited and ambiguous information about how to identify and intervene on psychosocial problems and unmet needs of breast cancer patients in the clinical settings in Korea [8, 9]. According to a recent study conducted with Korean breast cancer patients, patients reported high needs for counseling or having someone whom they could talk about psychosocial problems, but health professionals have difficulties to identify needs and to provide appropriate psychosocial care to cancer patients due to limited time and resources [7]. As a result, health care providers often fail to provide appropriate psychosocial care. In particular, non-interactive, standardized education that ignores individual differences in lifestyle and condition has resulted in ineffective outcomes and dissatisfaction among breast cancer survivors. There is a need for programs to train healthcare professionals to provide realistic and beneficial psychosocial support to breast cancer survivors [10].

Considering that effective communication and psycho social care training in cancer care can reduce patients’ emotional stress and uncertainty and can improve patient outcomes [11, 12], the Cancer Education Center (CEC) at Samsung Medical Center (SMC), in collaboration with the Korean National Cancer Center (KNCC) and the Korean Breast Cancer Society (KBCS), launched the BRAVO (Be Remarkable, Awesome, Vivid, and Optimistic) project in May 2012. The project is intended to help breast cancer survivors overcome the psychosocial challenges present both during and after cancer treatment. The project consisted of three parts: (1) training of trainers, (2) health education material development, and (3) community education. This paper will focus on the development and implementation of the Training of Trainers (TOT) program, called the BRAVO Navigator Training Program (BRAVO-NTP). The objective of the BRAVO-NTP was to provide healthcare professionals with the opportunity to develop skills and competencies for providing psychosocial support to breast cancer survivors. The purpose of this paper is to describe the development, implementation, and evaluation process of the BRAVO-NTP.

## Methods

### Setting and organization

The BRAVO-NTP was developed and implemented at the CEC. The CEC opened in 2008 to provide informational, psychological, and psychosocial support to cancer patients, their families, and the community. Monday through Friday, the CEC provides three core services to patients and families: (1) print and multimedia information materials, (2) interactive education (Wellness Program) for helping patients manage psychological distress, and (3) counseling services provided by oncology nurses. On average 150 to 200 cancer patients and families visit the CEC every day, with 30–50 participating in the Wellness Program.

### Program development

**Fig. 1:**
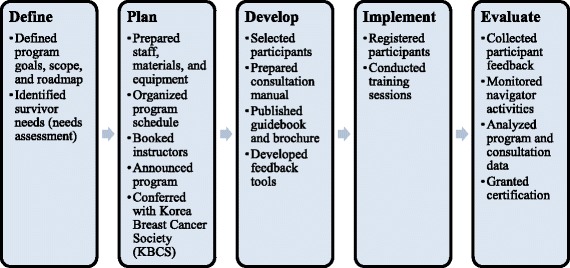
Development, implementation, and evaluation process of the BRAVO-NTP

The BRAVO-NTP development process consisted of six steps: (1) a needs assessment based on a literature review, expert meetings, a cross-sectional survey, and qualitative interviews; (2) defining the scope of the program by establishing program goals, training objectives, and a roadmap; (3) selecting educational methods and techniques best suited for achieving the objectives; (4) developing program content including training manuals and educational materials; (5) implementing the program; and (6) evaluating our performance (Fig. 1). The following section describes how each step was operationalized.

#### Step 1. Needs assessment

To define a clearly established goal for the program, we performed a detailed literature review, held three expert discussion meetings, and developed a mixed-methods needs assessment. To determine the unmet psychosocial needs of breast cancer survivors, we conducted a cross-sectional survey followed by qualitative interviews. For the cross-sectional survey, we questioned patients who visited the breast cancer centers of Samsung Medical Center (SMC) and the Korean National Cancer Center (KNCC) from of August 23th to 30th, 2012. Patients were eligible to respond to the survey if they were diagnosed with stage 1–3 breast cancer more than 1 year prior, and if they had no sign of recurrence or metastasis at the time of the survey. We asked participants regarding 60 most frequently reported psychosocial challenges of breast cancer survivors. The item is composed of a 4-point Likert scale (1 = no difficulty, 4 = very difficult) and 5 multi-item challenges domains that included (1) fear of recurrence (10 items), (2) physical and psychological symptoms (16 items), (3) family and social roles (19 items), (4) workplace (9 items), and (5) life planning (6 items). Patients reported that ‘fear of cancer recurrence’, ‘health management’, and ‘physical-psychological symptoms’ as the top three unmet needs regardless of survival length. Patients with survival duration less than 2 years had higher level of unmet needs in the insufficient information, changes in appearance, cancer stigma, sexuality and getting back to work domain. Patients with a survival duration of 2–5 years had higher unmet needs than patients in the re-entry group (survival duration less than 2 years) in overall life burden, married woman role, difficulties in life planning, and managing relationship domains. Detailed results of the needs assessment are included in a separate paper (Additional file 1).

Further we conducted semi-structured in-depth interviews with 30 breast cancer survivors to identify specific psychosocial challenges experienced during survivorship from September 2012 to March 2013 (Additional file 2). Through these interviews, we were able to acquire details about the specific physical, psychological, and social challenges that degrade the quality of life of breast cancer survivors both during and after treatment. Most of survivors described ‘fear of cancer recurrence’ as a cause of indefinite anxiety which gets worst during re-entry period. Survivors reported that many re-entry concerns were often triggered by insufficient information needs in fear of cancer recurrence. In addition, working survivors experienced additional emotional burdens due to cancer stigma at work place. Married women expressed challenges with multiple roles and responsibilities in the family or communication problem with families after cancer. We used this information to develop educational curricula and program content. Both the cross-sectional survey and the qualitative interview study were approved by the Institutional Review Boards (IRB) of SMC and KNCC.

#### Step 2. Training objectives

The results of the needs assessment were discussed by the BRAVO-NTP advisory board, which consisted of two family medicine physicians, two oncologists, and two oncology nurses. To achieve the overall aim of the BRAVO-NTP, we used the results of the needs assessment to formulate specific objectives designed to improve healthcare providers’ knowledge, skills, and competencies for providing psychosocial support to breast cancer survivors.

#### Step 3. Methods and educational material development

With the guidance of the advisory board, we developed training methods and educational materials in accordance with program objectives. Considering time and logistic limitations, we decided to adopt a two-day intensive training schedule as opposed to a more typical five-day schedule. Education materials and counseling notes were developed to maximize the effect of training and to ensure the availability of practical support solutions for survivors.

#### Step 4. Program content

Program content was determined based on the results of the needs assessment and the advisory meetings. We strived to include topics that focused on the unmet needs of breast cancer patients. While the BRAVO-NTP was primarily focused on psychosocial support, the advisory board recommended the inclusion of physical health topics, citing the strong correlation between physical and psychosocial well-being. The result was the development of a comprehensive health program for breast cancer survivors. Next, the team invited advisory board-recommended experts to help develop the specific content required for lectures and training materials. All program content was carefully discussed and articulated by these experts in collaboration with the BRAVO-NTP team. Additionally, a training manual was developed in accordance with program content.

#### Step 5. Implementation

The BRAVO-NTP was implemented in cooperation with the KBCS and the Korean Oncology Nursing Society (KONS) by advertising to major breast cancer clinics throughout Korea from June 17th to August 5th 2013. A total of 44 nurses and social workers from 24 hospitals participated in the two-day intensive training from August 23rd to 24th 2013. After the training, participants were asked to counsel their own patients for a month and to send their counseling notes and patients’ satisfaction responses to the program office. They were able to receive certification of the BRAVO-NTP once they submitted more than three counseling notes. BRAVO-NTP was approved by the IRB at SMC. Informed consent was obtained from each participant before they received the training.

#### Step 6. Evaluation

To measure the quality and effect of the training, attendees were asked to evaluate their overall satisfaction with the program following the two-day intensive training. Evaluations measured both the educational curriculum (program content, training manual) and the training environment (venue, snacks and meals, program organization). Satisfaction was measured using a five-point Likert scale, with higher scores indicating greater satisfaction.

To evaluate the impact of the training program on clinical practice, participants were asked to practice the skills and knowledge they learned from the training program and submit at least 3 counseling cases with actual patients within 3 months after the training. With each counseling case, participants were asked to mark the educational materials and resources they used from the training. In addition, we asked all participants how much the training was helpful for providing psychosocial support to patients and whether they agreed to use educational materials and resources obtained from the training in the 3 month period after the training. Both questions were asked with a 5 point Liker’s scale (0 means not at all and 4 means very much) and a higher score means more satisfaction and willingness to use the materials in the future.

## Results

### Needs assessment

A total of 298 patients participated in the needs assessment survey. Among them, 113 patients were excluded from the final analysis as they had been diagnosed less than 1 year prior, resulting in 185. According to the survey, fear of recurrence was the greatest concern among breast cancer survivors. The top three concerns in each domain are presented in Table 1.

**Table 1:** Top three concerns determined by the needs assessment (*N* = 280)

Domains	Problems^a^	*N* (%)
Fear of recurrence	Fear of recurrence or metastasis	244 (87.1)
	Fear of second cancer	241 (86.1)
	Fear of regular surveillance	216 (77.1)
Physical and psychological symptoms	Physical fatigue	235 (83.9)
	Loss of memory	218 (77.9)
	Psychological distress	204 (72.9)
Family and society	Worrying about children’s cancer risk	188 (67.1)
	Feeling sorry for the family	175 (62.5)
	Difficulties with sexual life	155 (55.4)
Work place	Decreased work abilities	174 (62.1)
	Reluctance to disclose cancer to others	156 (55.7)
	Stigma toward cancer survivors	128 (45.7)
Life planning	Difficulty keeping healthy lifestyles	181 (64.6)
	Difficulty planning for the future	146 (52.1)
	Difficulty accomplishing daily activities	146 (52.1)

^a^Order by rank

In accordance with previous research, fatigue was reported as the most significant physical and psychological symptom of breast cancer patients. Following by significant symptoms were memory loss and distress.

Regarding family and social issues, most patients were worried that their children would inherit breast cancer. Additionally, over half of the respondents expressed guilt about having breast cancer as it disrupted family life and caused sexual difficulties with their partners.

In the work place, about two-thirds of working patients experienced decreased work performance and more than half were reluctant to disclose their condition to their colleagues.

Regarding general health and well-being, most patients expressed difficulty maintaining healthy lifestyles and planning for the future.

### Training objectives

Based on the needs assessment and expert recommendations, the BRAVO-NTP team established an overall objective for the program: to improve health professionals’ knowledge and skills to provide appropriate supportive care to breast cancer patients to overcome psychosocial problems during survivorship. The first specific objective of the BRAVO-NTP was to explain the problems and difficulties breast cancer survivors face following treatment. The second specific objective was to teach skills to assess survivors’ physical and psychosocial status (focusing on unexpressed psychosocial issues) and understanding survivors’ primary concerns. The third specific objective was to improve communication skills and provide appropriate resources and services.

### Methods and education material development

Most training sessions (with the exception of Session Six: Developing Communication Skills) were run as lectures with interactive question and answer sessions. Session Six entailed a small group exercise consisting of a case review and counseling practice. A training manual, counseling notes, and a brochure were developed to support the training. The training manual included general information about the psychosocial problems of breast cancer patients, a guideline for counseling, and lecture notes from each training session. The lecture notes consisted of five parts: (1) problems with transition, (2) comprehensive survivorship care, (3) issues with family, (4) going back to the community, and (5) developing communication skills with the checklists.

A counseling note was developed for navigators describing tools to assess patients’ physical and psychological status. The note is composed of two parts. The first part is “Patient Self-Assessment,” which asks patients to describe the difficulties in their daily life, their stress levels, and any menopause symptoms within the last month. The second part helps navigators to assess the current treatment and health status of patients, health habits, and any concerns or problems patients express during counseling. Training materials also included a 36-page brochure about post-treatment management which the navigator could provide to patients during and after counseling (Appendix A). The brochure provides useful guidelines about the management of physical difficulties such as menopause symptoms, neuropathy, and stress. It also provides tips for a healthy lifestyle including exercise and diet recommendations as well as advice on how to monitor for recurrent or secondary cancer following treatment.

In addition, a book and film were developed for the BRAVO project and shared with participants. The book, entitled “BRAVO for Your Life!” was based on the inspiring stories of breast cancer survivors and shared their tips for success. The book also included information for families, friends, and coworkers.

Considering that movie enhances a narrative model framed emotions and that audiovisual support is a powerful resources in teaching [13], we made a short film to help participants understand what kinds of psychosocial challenges breast cancer survivors and family member face every day. The film, entitled “Smile Again,” was inspired by the true stories of two women’s battle with breast cancer and how they coped with the difficulties of their cancer journey, especially their transition from patient to survivor. The film’s epilogue contains the voices of four survivors as they share their stories and give advice for future patients and their families. The film can be found here: (https://www.youtube.com/watch?v=bo4f7NWbUBk&t=11s).

### Program content

The BRAVO-NTP included a total of 16 lectures held over the course of seven sessions. Contents and a detailed schedule are presented in Table 2.

**Table 2:** Schedule of the BRAVO Navigator Training Program

Specific lecture contents
Introduction
Breast cancer survivorship in Korea
Introduction to the BRAVO Navigator Training Program
Session 1: Problems with transition
Management of physical symptoms
Sleep disorder, fatigue, concentration, neuropathy, pain, and lymphedema
Management of psychological symptoms
Fear of recurrence, depression
Session 2: Comprehensive survivorship care I
Care after treatment
Regular surveillance, vaccination, secondary cancer prevention
Health management during survivorship
Bodyweight, diet, exercise, skin and dental care
Understanding alternative and complementary therapies
Management of menopause symptoms
Watch the film “Smile Again”
Share stories of breast cancer survivors
Session 3: Comprehensive survivorship care II
Distress management
Sexuality
Fertility and pregnancy
Session 4: Family
Communication with family: Hereditary risk
Session 5: Community
Support for vulnerable social groups
Living in a community
Cancer stigma and social discrimination, returning to work
Session 6: Developing communication practices
Principles and techniques of counseling
Practice for counseling cases

The training program spreads over 2 days. On the first day, lecture contents included: (1) an introduction to the BRAVO-NTP; (2) problems with transitioning from patient to survivor; and (3) comprehensive survivorship care including health management, alternative and complementary therapies, and menopause.

During the evening program of the first day, participants had an opportunity to share thoughts and emotional sympathy with breast cancer patients and survivors. Before the session began, participants watched the film “Smile Again,” which dealt with the challenges of breast cancer patients. Furthermore, we invited two survivors, a single woman in her 30s and a married working woman in her 40s, to share their experience about how breast cancer affected their family, work, and social life. By watching the film and sharing stories with breast cancer survivors, participants had the opportunity to understand the diversity of problems facing survivors.

On the second day, lectures focused on (1) comprehensive survivorship care including distress management, sexuality, fertility, and pregnancy; (2) family; (3) community; and (4) practice.

The following is a detailed breakdown of session content:

0. Introduction Session

Prior to beginning the training sessions, the group discussed the current status of breast cancer survivors in Korea, and explored the need for trained specialists to support breast cancer survivors.

1. First Session (2 h)

The session began by describing the effective management of the physical and psychosocial difficulties breast cancer survivors face following treatment. These difficulties include fatigue, memory loss, pain, lymphedema, fear of recurrence, and depression. Lectures were delivered by psychology professionals with extensive experience dealing with post-treatment symptoms.

2. Second Session (4 h)

The second session focused on comprehensive survivorship care (CSC), which is intended to improve breast cancer survivors’ quality of life. Due to limited time, CSC was presented over the course of 2 days as Part I and II. Part I began by promoting good physical health practices such as regular exams, secondary cancer prevention, and vaccinations for family members. Next, the session focused on daily activity management such as maintaining a healthy weight, eating nutritious food, and determining suitable exercise methods. Specialists in complementary and alternative medicine (CAM) were invited to identify the appropriate CAM therapy for survivors. The session concluded with instruction on how to manage menopause symptoms to improve the effects of hormonal therapy and overall quality of life. This session focused on practical methods based on scientific knowledge as opposed to existing education which focused primarily on theoretical concepts.

After second session, participants watched the film “Smile Again.” By watching the film, participants were able to better understand the impact of breast cancer on patients’ and families’ psychosocial well-being with a perspective that they may have not obtained from clinical experience.

3. Third session (2 h)

This session presented Part II of CSC which included methods to manage distress, sexual issues, pregnancy, childbirth, and breastfeeding associated with cancer diagnosis.

4. Fourth session (1 h)

Based on the needs assessment, we determined that communication with family is a major problem among breast cancer survivors. An advanced nurse taught the necessary skills to understand and effectively cope with family concerns. This session also included information about genetic issues related to breast cancer.

5. Fifth session (1 h)

A trained social worker discussed ongoing breast cancer studies including government policies for vulnerable women with breast cancer and coping with the stigma associated with the disease.

6. Sixth Session (2 h)

The final session aimed to practice and strengthen realistic counseling skills. Lessons were reinforced by a psychology physician present during practical exercises.

### Implementation

A total of 41 nurses and three social workers participated in the BRAVO-NTP (Table 3). All participants were female between the ages of 27 and 52 (37.5 ± 6.4) and a majority held a bachelor’s degree (64.3%). The mean years of participants’ clinical experience was 11.1 (± 5.6), with the mean years of oncology-specific experience being 8.1 (± 5.2). Most participants had worked in the capital city, Seoul.

**Table 3:** Demographic characteristics of participants (*N* = 42)

Characteristics	Mean ± SD / Range or *N* (%)
Age	37.5 ± 6.4 / 27–52
Female	42 (100.0)
Education status	
College (associate degree)	6 (14.3)
University (bachelor’s degree)	27 (64.3)
Graduate school (master’s degree)	9 (21.4)
Years of clinical experience	11.1 ± 5.6 / 3.5–26
Years of oncology-related experience	8.1 ± 5.2 / 0.5–24
Work location	
Capital city	28 (66.7)
Metropolitan or rural	14 (33.3)

The duration of the training program was 2 days (21 h total) from August 23rd to 24th 2013. Most sessions consisted of lectures and presentations while some programs, such as “Communication with Family,” used a variety of learning methods including videos, discussions, and group exercise.

The BRAVO team developed a training manual, film, and book, which were given to participants to enhance their skills. After the trainees completed the course, additional resources such as counseling notes and a brochure were supplied in order to maintain the training effect of the BRAVO-NTP.

Following completion of the program, we granted KBCS-accredited certification to those participants who provided education and consultation to a minimum of three breast cancer survivors. Among the 44 participants of the course, 31(70.4%) have been certified as navigators after they submitted counseling notes documenting their consultation cases.

### Evaluation

The program was evaluated on both the overall organization and the individual lectures (Table 4). Using a survey form, most participants reported satisfaction with the training program. Participants rated the course using a five-point Likert scale, with higher scores indicating greater satisfaction.

**Table 4:** Evaluation of lecture contents (*N* = 39)

Lecture contents	Score
Introduction	
Breast cancer survivorship in Korea	3.40
Introduction to the BRAVO Navigator Training Program	4.41
Problems with transition	
Management of physical symptoms	4.87
Management of psychological symptoms	3.69
Comprehensive survivorship care	
Care after treatment	4.38
Health management during survivorship	3.93
Understanding alternative and complementary therapies	3.82
Management of menopause symptoms	4.28
Distress management	4.17
Sexuality	4.26
Fertility and pregnancy	4.44
Family	
Communication with family	3.89
Community	
Support for vulnerable social groups	3.44
Living in a community	4.25
Practice	
Principles and techniques of counseling	3.49
Practice for counseling cases	3.00
Average	3.98

Regarding the overall operation of the course, participants responded as follows: program contents (4.04), trainee guidebook (3.82), location and environment (4.10), meals and snacks (4.23), and program organization (4.19). The most highly rated lecture was “Management of Physical Symptoms” (4.87) while “Practicing for Counseling Cases” (3.00) was rated lowest.

Participants also provided written feedback regarding their overall evaluation of the program. Requests included more practical information about finance, sexuality, and psychological concerns. They also wanted simpler assessment tools for more effective consultation. Table 5 shows select feedback quotes from participants.

**Table 5:** Feedback quotes from participants

Feedback content
“Almost all survivors know how to deal with psychosocial issues after treatment, because they have already spent a lot of time agonizing over their adjustment.”
“I think that the next program needs to be enhanced education about how to help their financial problems, insurance, and psychological support.”
“I need more realistic information about complementary and alternative therapies.”
“How can I begin to approach patients who have concerns about sexual problems and genetic issues?”
“We need simpler assessment tools before consultation.”

In terms of the impact of the training on clinical practice, 29 (69.1%) nurses among 42 participants submitted 85 counseling cases. Most common used resources for counseling were about distress management, management of physical symptoms, and management of psychosocial symptoms followed by communication with family. Of total, 58.6% said that the training was helpful or very helpful for providing psychosocial support to patients, and 89.7% agreed or strongly agreed to use the provided education materials and resources in the future.

**Fig. 2:**
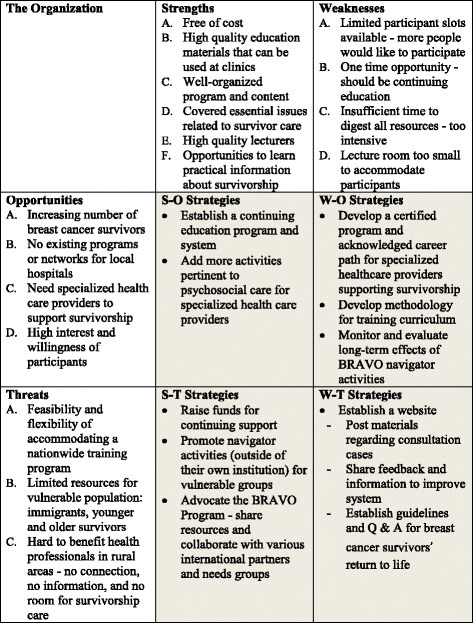
SWOT analysis

Even though the BRAVO-NTP was designed to provide guidelines and a methodical approach to enhancing the role of the navigator, some practical weaknesses became evident following the BRAVO-NTP’s conclusion. In particular, a major issue was related to the continuity of the program. Based on feedback and evaluations, the program needs to be delivered as continuous, regular education to maximize effectiveness. Based on the results of the strengths, weaknesses, opportunities, and threats (SWOT) analysis [14], recommendations for future BRAVO programs appear in Fig. 2.

## Discussion

Due to increasing survival rates for breast cancer patients, healthcare professionals need to understand the diverse challenges facing breast cancer survivors [15]. Accordingly, Korea has joined the global trend of enlisting healthcare professionals to provide comprehensive and coordinated care for survivors [16]. While survivorship clinics and comprehensive programs have been established in countries such as the U.S., similar support is limited in Korea [15]. Consequently, healthcare professionals in Korea are unprepared to provide survivorship care, necessitating tailored education [10, 16] and specific information to support ongoing medical and psychological care [17].

Based on the needs of breast cancer survivors, we developed the BRAVO-NTP to provide nationwide complementary training education for healthcare professionals tasked with providing psychosocial and physical care to breast cancer patients. The two-day education program focused on training a well-qualified navigator to guide breast cancer survivors to improved quality of life through individualized consultation both during treatment and beyond.

The challenge of developing a comprehensive training program to support breast cancer survivors is complicated by several factors. First, the period of “survivorship” is not well defined [3]. Long-term survivorship could be anything from living beyond the post-reentry phase (12 to 18 months after treatment) to living 5 years without recurrence [3, 18]. Unclear time boundaries complicate the provision of appropriate care [3]. In addition, the lack of evidence-based research and consensus limits the ability of healthcare providers to apply evidence-based practice guidelines for a broad spectrum of issues faced by breast cancer survivors [15]. Furthermore, healthcare professionals lack clear guidelines to support survivorship care. As a solution, comprehensive, coordinated, and ongoing training should be available to professionals who lack the information and knowledge necessary to implement survivorship care [15, 19–22].

In Korea, a lack of consensus regarding the essential elements of survivorship care for breast cancer patients forms a barrier to the delivery of optimal care [15]. Adequate survivorship care should be modified based on the individual needs of patients, such as culture and life stage [23, 24]. This is consistent with our study, which showed that breast cancer survivors preferred the individualized components of the program [23]. Core elements of a survivor counseling program should include guidance for the long-term effects of cancer treatment, monitoring for recurrence, secondary cancers, psychosocial issues, health promotion (e.g. immunization), diet, exercise, and communication with healthcare providers [16].

Though the topics in our study are similar to those recommended by the Institute of Medicine [16], we would add that the needs assessment should be updated regularly to account for the evolving needs of patients. For effective updates to occur, ongoing counseling and experience pertinent to survivorship care is necessary. An effective needs assessment must account for the perspectives of both survivors and healthcare providers [7, 16]. In addition, we recognize that our program is restricted by several factors. Due to logistic constraints, our program contents could not include all challenges faced by breast cancer survivors. Instead, we focused on the essential components of survivorship care that could best serve the needs of a trainer education program.

In our BRAVO-NTP, training sessions focused on psychological issues (e.g. “Support for Vulnerable Social Groups” and “Management of Psychological Symptoms”) received relatively lower satisfaction by participants. This might be because the participants were unfamiliar with benefits of counseling and communication. Although psychosocial care could help survivors to know what to expect following treatment and teaches them to effectively deal with the complex problems presented by cancer treatment [3], psychosocial issues are often given a lower priority compared to other medical issues in oncology care settings [15]. Also, it psychosocial support and counseling might require long-term training and practice as opposed to the short-term training offered by the program.

The SWOT analysis of the BRAVO-NTP suggests a program of continuing education would be superior to short, intensive training. A possible strategy could be the development of a website providing updated information for healthcare providers [15]. The site could include educational materials regarding consultation cases as well as evidence based information for physical and psychosocial management [15]. The site could also host web-based educational modules and information summaries with evidence-based guidelines, which could help mitigate the logistic limitations of an intensive on-site training program [15, 25]. Additionally, a question and answer (Q and A) session could elicit feedback from healthcare professionals including valuable information and potential improvements to systematic care. The site could also provide tools such as printable checklists and guidelines as healthcare providers prefer paper-based documentation for specific information [25]. These strategies could help healthcare providers ensure the continuity of optimal care for breast cancer survivors [26–28].

Feedback also indicated the BRAVO program could benefit by focusing on practical guidelines for topics such as sexuality, finance, and psychosocial issues. Additionally, we should develop simpler and more useful assessment tools (e.g. a breast cancer journey passport) as opposed to the 20-page consultation note disseminated by the first BRAVO program. We also identified a need for psychosocial intervention methods for survivors in the re-entry phase rather than survivors who are several years beyond treatment. Thus, we would set out to develop an intervention designed to address the unique psychosocial problems of women just finishing treatment and attempting to return to normal life.

## Conclusion

The BRAVO-NTP was a comprehensive and coordinated educational program designed to train healthcare professionals to implement survivorship care, emphasizing psychosocial support, based on the needs assessment of breast cancer survivors. However, to maximize effectiveness, the program should continuously evolve along with the needs of breast cancer survivors. Future plans for the program include the augmentation of practice sessions with psychosocial counseling.

The program was intended as continuing education for healthcare providers who focus on breast cancer survivorship care. To support further education, a website with various educational materials could be developed based on the contents of the program. In addition, the program could provide training and education for graduate nursing students as they prepare to become specialized oncology nurses.

## Additional files

Additional file 1: Needs assessment for breast cancer survivors. Questions used for needs assessment of breast cancer survivors. (DOCX 26 kb)

Additional file 2: Semi-structure interview guidelines. Semi-structure interview guidelines for finding details of difficulties breast cancer survivors experience during re-entry period based on the results of the quantitative survey. (DOCX 20 kb)

## Abbreviations

BRAVO: Be remarkable, awesome, vivid, and optimistic
BRAVO-NTP: BRAVO Navigator Training Program
CAM: Complementary and alternative medicine
CEC: Cancer Education Center
CSC: Comprehensive survivorship care
KBCS: Korean Breast Cancer Society
KNCC: Korean National Cancer Center
KONS: Korean Oncology Nursing Society
SMC: Samsung Medical Center
SWOT: Strengths, weaknesses, opportunities, and threats
TOT: Training of trainers
