# Supplementary material for: A train the trainer program for healthcare professionals tasked with providing psychosocial support to breast cancer survivors
Source: BMC Cancer. 2018 Jan 6;18:45. doi: 10.1186/s12885-017-3965-2 (PMC5756444; doi:10.1186/s12885-017-3965-2)
Supplement: Supplementary file 1 — Needs assessment for breast cancer survivors. Questions used for needs assessment of breast cancer survivors. (DOCX 26 kb) [file 12885_2017_3965_MOESM1_ESM.docx]

- **Needs Assessment for Breast Cancer Survivors**

**We are interested in difficulties during survivorship. Please answer all of the questions yourself by circling the number that best applies to you. There are no "right" or "wrong" answers. The information that you provide will remain strictly confidential.**

|  | Problem list | Not at all | No | Somewhat difficult | Very difficult |
| --- | --- | --- | --- | --- | --- |
| **1) Fear of recurrence;** | | | | | |
| 1 | Fear of tests of the regular surveillance | 1 | 2 | 3 | 4 |
| 2 | Fear of having a second cancer | 1 | 2 | 3 | 4 |
| 3 | Fear of recurrence or metastasis | 1 | 2 | 3 | 4 |
| 4 | Not know about overall health management methods | 1 | 2 | 3 | 4 |
| 5 | Know health management methods but difficult to apply or follow | 1 | 2 | 3 | 4 |
| 6 | Not know appropriate diet methods. | 1 | 2 | 3 | 4 |
| 7 | Know how to diet but difficult to apply or follow | 1 | 2 | 3 | 4 |
| 8 | Lack of information about dietary supplement | 1 | 2 | 3 | 4 |
| 9 | Not know appropriate exercise methods | 1 | 2 | 3 | 4 |
| 10 | Know appropriate exercise methods but difficult to apply or follow | 1 | 2 | 3 | 4 |
| **2) Physical and psychological symptoms** | | | | | |
| 11 | Lymph edema | 1 | 2 | 3 | 4 |
| 12 | Physical fatigue | 1 | 2 | 3 | 4 |
| 13 | Psychological fatigue | 1 | 2 | 3 | 4 |
| 14 | Loss of memory | 1 | 2 | 3 | 4 |
| 15 | Sleep disorder (Somnipathy) | 1 | 2 | 3 | 4 |
| 16 | Decreased ability of concentration | 1 | 2 | 3 | 4 |
| 17 | Altered appearance (hair loss, skin change, body shape) | 1 | 2 | 3 | 4 |
| 18 | Difficulties to choose clothes due to breast surgery | 1 | 2 | 3 | 4 |
| 19 | Difficulty of wearing an artificial breast | 1 | 2 | 3 | 4 |
| 20 | Loss of self-confidence | 1 | 2 | 3 | 4 |
| 21 | Menopause symptoms (hot flashes, heart palpitation) | 1 | 2 | 3 | 4 |
| 22 | Want to have baby but keep failing (infertility) | 1 | 2 | 3 | 4 |
| 23 | Pain during sex (Vaginal dryness) | 1 | 2 | 3 | 4 |
| 24 | Depression | 1 | 2 | 3 | 4 |
| 25 | Lack of energy(Lethargy) | 1 | 2 | 3 | 4 |
| 26 | Difficult to keep ideal body weight | 1 | 2 | 3 | 4 |
| **3) Family and social roles** | | | | | |
| 27 | Hurt by family without reason | 1 | 2 | 3 | 4 |
| 28 | Physician’ does not understand me | 1 | 2 | 3 | 4 |
| 29 | Friends do not understand me | 1 | 2 | 3 | 4 |
| 30 | No person I can talk about my hurt feelings | 1 | 2 | 3 | 4 |
| 31` | Losing interest in sex | 1 | 2 | 3 | 4 |
| 32 | Difficulties to join activities with friends | 1 | 2 | 3 | 4 |
| 33 | Financial difficulties | 1 | 2 | 3 | 4 |
| 34 | Pressure to be being positive against cancer | 1 | 2 | 3 | 4 |
| (Just for women who are married or living with partners) | | | | | |
| 35 | Difficulties for being a housewife | 1 | 2 | 3 | 4 |
| 36 | Difficulties for being a mother | 1 | 2 | 3 | 4 |
| 37 | Difficulties for being a wife | 1 | 2 | 3 | 4 |
| 38 | Responsibility of taking care of family. | 1 | 2 | 3 | 4 |
| 39 | Sorry feelings to the family | 1 | 2 | 3 | 4 |
| 40 | Relationship with in-laws | 1 | 2 | 3 | 4 |
| 41 | Husband cannot understand me | 1 | 2 | 3 | 4 |
| 42 | Family does not consider that I am cancer patients | 1 | 2 | 3 | 4 |
| 43 | Worry about children’s risk of getting cancer because of me | 1 | 2 | 3 | 4 |
| 44 | Children felt ashamed that mother has cancer | 1 | 2 | 3 | 4 |
| 45 | Husband’s/Partner’s affair | 1 | 2 | 3 | 4 |
| **4) Work place** | | | | | |
| 46 | Stigma towards cancer patients | 1 | 2 | 3 | 4 |
| 47 | Do not want to disclose cancer to others | 1 | 2 | 3 | 4 |
| 48 | Feel uncomfortable with attention from coworkers | 1 | 2 | 3 | 4 |
| 49 | Reduced work ability | 1 | 2 | 3 | 4 |
| 50 | Need to keep working due to financial reason (want to rest) | 1 | 2 | 3 | 4 |
| 51 | Getting a job (discrimination because of cancer) | 1 | 2 | 3 | 4 |
| 52 | Returning to work (social gathering | 1 | 2 | 3 | 4 |
| 53 | Losing opportunities for promotion | 1 | 2 | 3 | 4 |
| 54 | Schoolwork | 1 | 2 | 3 | 4 |
| **5) Life planning** | | | | | |
| 55 | Not know well about distress management | 1 | 2 | 3 | 4 |
| 56 | Know distress management methods but difficult to apply/follow | 1 | 2 | 3 | 4 |
| 57 | Feel lost (not clear about future) | 1 | 2 | 3 | 4 |
| 58 | Difficult to plan future | 1 | 2 | 3 | 4 |
| 59 | Difficult to achieve things in daily activities | 1 | 2 | 3 | 4 |
| 60 | Difficult to accept being different (before and after treatment) | 1 | 2 | 3 | 4 |
