# Supplementary material for: A train the trainer program for healthcare professionals tasked with providing psychosocial support to breast cancer survivors
Source: BMC Cancer. 2018 Jan 6;18:45. doi: 10.1186/s12885-017-3965-2 (PMC5756444; doi:10.1186/s12885-017-3965-2)
Supplement: Supplementary file 2 — Semi-structure interview guidelines. Semi-structure interview guidelines for finding details of difficulties breast cancer survivors experience during re-entry period based on the results of the quantitative survey. (DOCX 20 kb) [file 12885_2017_3965_MOESM2_ESM.docx]

- **Semi-structure interview guidelines**

**We are interested in difficulties during survivorship. We are going to share with you the results of our quantitative survey. Please tell us what specific problems or situation you experience regarding each topic, how you tried to overcome those challenges, and what was the most effective or helpful for you to overcome difficulties during survivorship.**

**1. Most of the survivors said that they had problems with “fear of recurrence.” Can you tell me when you experience “fear of recurrence” and how it interfere your daily life?**

**How did you handle them? Do you have any recommendations to other survivors?**

**2. Survivors mentioned that they kept having physical and psychological symptoms even few years after treatment. Can you tell us what physical or psychological symptoms bother you most? How did you handle them? Do you have any recommendations to other survivors?**

**3. Some married women expressed challenges with multiple roles and responsibilities in the family or communication problem with families after cancer. Can you tell us your experience? What were the most challenging in terms of your family and social roles? How did you handle them? Do you have any recommendations to other survivors?**

**4. Did you work when you were diagnosed with breast cancer? According to our survey, many working survivors experienced additional emotional burdens due to cancer stigma at work place. Can you tell us your experience? What were the most challenging when you returned to work? How did you handle them? Do you have any recommendations to other survivors?**

**5. Many of survivors in our quantitative survey, they felt lost and have problems to plan their life after cancer. Have you ever had similar experience? Can you tell us your experience? How did you handle them? Do you have any recommendations to other survivors?**

**6. Besides things we have discussed, if you have any, can you tell us problems or difficulties you had during survivorship? Can you tell detailed information about it? When and how? How did you handle them? Do you have any recommendations to other survivors?**

**7. We are going to make an education booklet for survivors during re-entry period, what information do you think that we should include.**

**Thank you for your time.**
